# Clinical trials of surgical neuromodulation therapies for epilepsy: Past, present & future

**DOI:** 10.1016/j.neurot.2026.e00935

**Published:** 2026-05-29

**Authors:** Rory J. Piper, Martin M. Tisdall

**Affiliations:** a Developmental Neurosciences, UCL Great Ormond Street Institute of Child Health, University College London, 30 Guilford Street, London, WC1N 1EH, UK; b Department of Neurosurgery, Great Ormond Street Hospital, London, WC1N 3JH, UK

**Keywords:** Epilepsy, Neuromodulation, Neurostimulation, Clinical trials

## Abstract

Neuromodulation is expanding as an avenue of therapy for patients with either focal- or generalized-onset epilepsy. Current implantable surgical options include deep brain stimulation (DBS), responsive neurostimulation (RNS®; NeuroPace), vagus nerve stimulation (VNS) and, more recently, subscalp, transcranial stimulation (EASEE®). This review of the literature discusses the milestone clinical trials that have shaped our practice of surgical neuromodulation for epilepsy, summarizes ongoing clinical trials, and speculates on the future of clinical trials in this field.

## Background

Epilepsy is a neurological disorder that predisposes those affected to have epileptic seizures – the symptoms of abnormal, excessive, or synchronous electrical brain activity [1]. Epilepsy is a common condition – it affects approximately 1 in 100 people and over 65 million people worldwide [2]. Epilepsy and seizures have significant consequences for the wellbeing of patients – including a higher risk of injuries, hospital admissions, psychosocial problems, and premature death.

Although antiseizure medications (ASM) are effective in controlling seizures for many patients with epilepsy, approximately one-third will continue to have seizures and are considered to have *‘drug resistant epilepsy’* (DRE) [3]. A carefully selected group of patients with focal DRE are eligible for epilepsy surgery to resect the epileptogenic zone and provide seizure freedom [4,5]. However, not all patients with focal DRE are (a) eligible for resective epilepsy surgery; and (b) seizure free following epilepsy surgery. There is, therefore, a cohort of patients with either focal or generalized-onset DRE that requires an alternative, novel therapeutic approach.

Neuromodulation therapies are increasingly being adopted for the treatment of patients with either focal or generalized-onset seizures and DRE [6,7]. Neuromodulation therapies aim to disrupt the pathological seizure propagation network [8], reduce the burden of seizure frequency and severity, and ultimately improve quality of life for patients. Intracranial neuromodulation treatments currently include deep brain stimulation (DBS) and responsive neurostimulation (*RNS®, NeuroPace*). Surgical extracranial neuromodulation treatments include vagus nerve stimulation (VNS) and, more recently, the subscalp, transcranial EASEE® stimulation therapy for patients with focal DRE. In addition to providing a therapy to those without alternative options, compared to other resective surgery, neuromodulation therapies offer the added advantages of ‘minimally invasive’ surgeries and adjustable/reversible stimulation parameters, thus potentially offering a more flexible treatment option.

The purpose of this review is to summarize the milestone randomized controlled trials (RCT) that have shaped the field of surgical neuromodulation therapies for epilepsy. We then provide an overview of clinical trials currently registered and ongoing and then speculate on the future of clinical trials – providing an opinion on the significant needs for this field to move forwards. The clinical literature in neuromodulation for epilepsy is already vast and rapidly expanding, so we therefore focus on (a) long-term (‘chronic’ or implanted) surgical neuromodulation therapies; and (b) clinical trials (prospective interventional studies).

## Outcome measures in neuromodulation trials

The *Engel* [9] and *International League Against Epilepsy* (ILAE) [10] classifications of postoperative seizure freedom have been used in prior studies and clinical trials of resective epilepsy surgery, such as the landmark temporal lobectomy trial [11]. These categorical outcomes according to degrees to ‘*seizure freedom’* have been used to determine the ultimate success of the surgical interventions.

However, clinical trials of neuromodulation for epilepsy have not used seizure freedom as a primary outcome. Neuromodulation interventions have previously been referred to as *‘palliative’* (a term falling out of favour [12]) since the therapy is generally not intended, or likely, to achieve seizure freedom. Instead, neuromodulation therapies aim to provide patients with a ‘significant’ reduction in seizure burden. As will be described, the landmark clinical trials in neuromodulation for epilepsy have used *‘seizure frequency reduction’* as a statistical marker of measuring treatment efficacy. Some studies have binarized efficacy in neuromodulation according to whether a patient is a *‘responder’*, which is typically defined as a patient experiencing a seizure frequency reduction of more than 50% relative to their baseline.

The method of measuring seizure frequency reduction is key and deserves discussion, since trials and studies have used different approaches, which has implications for the interpretation and comparison of results. Patient/carer seizure diaries are a frequent method in clinical trials, which holds the advantages of being patient-reported and allowing each patient’s outcome data to be adjusted relative to their own specific baseline – allowing comparability across patients. Disadvantages of seizure diaries, however, include the laboursome experience for the patient/family (particularly when they are asked to count consecutive seizures for several weeks), potential for missing data from either non-compliance, lapses in record keeping, or seizures not being noticed (for example, nocturnal seizures or seizures without awareness). Seizure frequency may alternatively be measured on electroencephalography (EEG) recordings, which may provide more reliability in counting seizures, but are limited by the duration of the EEG, the complexity of attributing electrographic seizures to clinically experienced seizures, and deterioration of EEG recording quality over time. Alternatively, seizure counting or recording devices, including sub-scalp monitors [13] or wearables are increasingly being developed. Finally, some studies have asked patients/carers to provide an estimation on the seizures from a recent period, which poses a significant risk of recall bias and inaccuracy due to seizures experienced without awareness.

Other important outcome measures used in clinical trial reporting, usually as secondary measures include seizure severity (for example, the *Liverpool Seizure Severity Scale* in adults [14], or the *Hague Seizure Severity Scale* in children [15]), quality of life, or neuropsychological outcomes.

## Past clinical trials in neuromodulation for epilepsy

### Vagus nerve stimulation

VNS is an extracranial, implanted neuromodulation therapy for DRE. VNS is especially advantageous in its availability across different healthcare systems and across patient groups: it is utilised for patients with either focal- or generalized-onset seizures, and both adults and children with epilepsy. VNS involves the implantation of stimulating electrodes coiled around the vagus nerve (typically on the left side) and connected to an implanted pulse generator (typically in the chest wall). VNS delivers a rhythmical, duty cycle of stimulation (typically for 30 s at 5-min intervals), but patients are also able to activate the device using a magnet when seizure-onset is suspected, in an attempt to abort seizures. More recent models of VNS include an automated closed-loop stimulation algorithm triggered on sensing an ictal tachycardia [16]. Despite the therapy being available for several decades, the therapeutic mechanism remains poorly understood. Studies have suggested that the stimulation of the vagus nerve causes alterations in the ‘*vagus afferent network’*, which causes suppression of seizures via the connections of the nucleus solitarius and thalamus [17,18].

The landmark RCTs that have demonstrated the efficacy of VNS in epilepsy are first *The Vagus Nerve Stimulation Study Group* in 1995 [19] (which includes the patients reported in the study by Ben-Menachem et al. published in 1992 [20]), and the second by Handforth et al. in 1998 [21]. Both trials had a similar parallel RCT design that compared high-frequency (30 Hz) versus low-frequency (1 Hz; ‘sham’) vagus nerve stimulation in patients with focal-onset epilepsy (Table 1). Seizure frequency reduction was the primary outcome, but both trials also reported adverse effects and quality of life in subsequent studies.

**Table 1:** Comparison of the design and primary outcomes for the landmark clinical trials of vagus nerve stimulation (VNS) for epilepsy. ASM, antiseizure medication; ns, non-significant result; RCT, randomized controlled trial (RCT); VNS, vagus nerve stimulation.

	VNS Study Group 1995	Handforth et al. 1998
**Design**	RCT	RCT
**Randomization**	‘High’ versus ‘low’ frequency stimulation	‘High’ versus ‘low’ frequency stimulation
**Blinded parties**	Patients & an investigator measuring outcomes	Patients & an investigator measuring outcomes
**Number of patients**	114 randomized 54 vs. 60, respectively	198 randomized 94 vs. 103, respectively
**Follow-up**	12-weeks	3 months after 2-week ‘ramp-up’ period where stimulation maximized
**Key inclusion criteria** (not exhaustive)	- Drug-resistant focal-onset seizures. - At least six seizures per month during a 12-week preoperative period. - At least 12 years of age.	- Drug-resistant focal-onset seizures - At least six seizures with altered consciousness over 30 days, but no more than 21 days between seizures -12–65 yrs - Take 1–3 ASMs for >1 month or five ½ lives + 2 weeks prior to study enrolment
**Primary outcome**	Seizure frequency	Seizure frequency
**Mean seizure frequency reduction**	24.5% vs 6.1%, respectively (*p* = 0.01)	27.9% vs 15.2%, respectively (*p* = 0.04)
**Responder rate** (at least 50% seizure frequency reduction)	31 vs 13%, respectively (*p* = 0.02)	23.4% vs. 15.7%, respectively (ns)

Both trials showed that high-frequency VNS was superior to low-frequency VNS in providing seizure frequency reduction relative to baseline (24.5% and 27.9%, respectively) (Table 1). The Handforth trial also showed that high-frequency VNS was superior in decreasing the mean number of seizures with altered consciousness.

Although low-frequency stimulation was used to ‘protect the blinding’ process so that patients were aware of stimulation, it is noted that low-frequency stimulation also significantly caused a reduction in seizure frequency when compared to baseline. It is impossible to unpick any potential therapeutic effect of low frequency stimulation compared to the placebo effect that the device insertion (regardless of blinding to regime) would have had on self-reporting of seizure frequency. The follow-up duration (three-months) for these RCTs was short, and long-term efficacy was not addressed. However, follow-up open label studies have suggested that seizure reduction improves with duration of therapy [22,23].

The two RCTs discussed above recruited patients aged 12 years and older. An RCT by Klinkenberg et al. investigated VNS in children aged 3–17 years but interestingly did not show a statistically significant benefit of VNS in seizure frequency reduction [24]. A similar design of randomization of 41 children (with either focal or generalized-onset seizures) to either a high or low frequency VNS allocation was performed for 20 weeks, and it was found that 16% vs 21% were considered responders (50% or more seizure frequency reduction relative to baseline), respectively. However, the investigators concluded that VNS reduced seizure frequency by at least 50% in 26% of the patients in the subsequent open label phase and improved seizure severity. Despite these modest results, subsequent meta-analyses of over 8000 children calculated that responder rate was 63% at 24–48 months [23].

### Deep brain stimulation

DBS is an intracranial, implanted neuromodulation therapy that delivers electrical stimulation directly to key targets within the epileptogenic network to reduce the frequency and severity of epileptic seizures [6]. Trials of DBS have typically targeted brain regions that serve as a propagation waypoint in the epileptogenic network (most frequently in the thalamus) [8]. The ANT has received considerable attention as a target for focal-onset epilepsy therapy, the centromedian for generalized epilepsy therapy, and more recently the medial pulvinar is being explored (for example in Pizzo et al.’s PULSE Study [25]). However, there have also been studies of non-thalamic DBS – to directly target the epileptogenic zone for example, the hippocampus in bilateral temporal lobe epilepsy (TLE), motor targets, cerebellum and hypothalamus [26].

This section discusses the landmark trials in DBS in epilepsy – the Fisher et al.’s *‘SANTÉ’* study of anterior thalamic DBS for focal DRE [27], the Dalic et al.’s *‘ESTEL’* trial of centromedian thalamic DBS for *Lennox-Gastaut syndrome* (LGS), and the Suresh et al.’s *ADVANCE* trial of DBS for children with DRE [28] (summarized in Table 2).

**Table 2:** Landmark RCTs of intracranial neuromodulation therapies (deep brain stimulation (DBS) and responsive neurostimulation (RNS)) for epilepsy. ANT, anterior nucleus of the thalamus; ASM, antiseizure medication; CMT, centromedian nucleus of the thalamus; RCT, randomized controlled trial (RCT); RNS, responsive neurostimulation; VNS, vagus nerve stimulation.

Trial	Design	Primary objective	Key eligibility criteria (not exhaustive)	N	Method of seizure frequency recording	Device	Target	Primary outcome
Electrical stimulation of the anterior nucleus of thalamus for treatment of refractory epilepsy (SANTÉ) [27]	• RCT • Multi center (n = 17; USA) • Random allocation to active vs inactive stimulation	Comparison of seizure frequency reduction 3-months after implantation	- Adults (18–65 years) - Patients with focal-onset seizures - Seizure frequency ranging between 6/month to <10/day - ≥3 failed trials of different ASMs	54 vs. 55	Seizure diary	Medtronic kinetra DBS system	ANT (bilateral)	Median seizure frequency reduction was 40.4% in the stimulation group and 14.5% in the control group.
Electrical stimulation of thalamus for epilepsy of Lennox–Gastaut phenotype (ESTEL) [29]	• RCT • Single center (Australia) • Random allocation to active vs inactive stimulation	Comparison of seizure responder (>50% seizure frequency reduction) rates at 3 months	- Young adults and adults (15–65 years) - Lennox-Gastaut syndrome - Minimum pre-trial seizure frequency of ≥4 per month (28 days)	10 vs. 9	Seizure diary (also EEG as secondary measure)	Medtronic activa PC DBS system	CMT (bilateral)	Responder rate was 50% in the stimulation group and 22% in the control group
RNS pivotal study [30]	• Prospective • Multicentre (n = 32, USA) • Random allocation to active vs inactive stimulation	Comparison of seizure frequency reduction 12 weeks after implantation Safety (device related and device unrelated serious adverse events)	- Adults (18–70years) - Patients with focal-onset seizures - Trial and failure or at least 2 ASMs - At least average of 3 disabling seizures per month - Undergone ‘standard diagnostic testing’ that localized 1 or 2 seizure-onset zones	97 vs. 94	Seizure diary	Responsive Neurostimulation® (RNS) system (NeuroPace, mountain view, CA)	Epileptogenic zone	Seizure frequency reduction 37.9% in the stimulation group and 17.3% in the control group

The *‘Stimulation of the Anterior Nucleus of the Thalamus for Epilepsy’* (*SANTÉ*) trial marks the first RCT of DBS for adults with focal-onset epilepsy [27]. The *SANTÉ* trial was a prospective, multicenter, double-blind RCT comparing active vs inactive bilateral anterior thalamic nucleus DBS in adults with focal DRE. All participants received implantation of a *Medtronic Kinetra DBS system* and at one-month following surgery 110 patients were randomized and blinded to active vs inactive stimulation for three-months. Median seizure frequency reduction at three-months on seizure diaries was 40.4% in the stimulation group and 14.5% in the control group. At three-months, the end of the blinded period, there was no significant difference in 50% responder rates, seizure severity (Liverpool Seizure Severity Score) or quality of life scores.

Follow up, open-label studies of the SANT*É* cohort showed that median seizure frequency reduction improved to 56% at two years (n = 102), 69% (n = 74) at five years [57], and 75% (n = 73) and seven years [58]. The number of responders improved to 54%, 68% and 74%, respectively. Similarly, seizure severity and quality of life improved on follow-up studies. While these follow-up studies are of great importance in understanding the long-term efficacy, adverse events and device performance, the results should be interpreted with some caution. Firstly, there was a considerable dropout of patients toward the end of the follow up, and this may have included patients who responded poorly to DBS, although the authors did make some statistical compensation for this. Secondly, patients may have had ASM changes that altered the course of their seizure frequency independent of the effects of DBS. Lastly, a skeptical viewpoint would be that several years of seizure diary collection by patients and their carers would lead to fatigue and poor compliance, causing an apparent downward drift of seizure frequency. However, continual improvement in quality of life and seizure severity scoring would correlate with a reducing seizure frequency.

It is notable that 45% of the participants in *SANTÉ* had received previous VNS therapy and it is inferred that the *SANTÉ* cohort is a group of patients who are extremely difficult to treat. Firstly, this should be remembered when interpreting the implications of the trial results, but also raises questions as to whether DBS would be even more effective for patients with ‘less refractive’ epilepsy?

The *‘Electrical Stimulation of Thalamus for Epilepsy of Lennox–Gastaut phenotype (ESTEL)’* trial was a prospective, double blind RCT of DBS of the centromedian nucleus of the thalamus (CMT) to treat young adults (17–37 years) with LGS. Three months following implantation, patients were randomized to receive either stimulation ‘on’ or stimulation ‘off’ and head-to-head outcomes were compared after 3-months. After the blinded phase, a further three-month unblinded phase was completed. The primary outcome was the proportion of responders (≥50% reduction in seizures compared to baseline, recorded via seizure diaries) in the last month of the blinded comparison phase of the trial. The result was that 5/10 patients in the ‘on’ group versus 2/9 patients in the ‘off’ group were responders. Although this was not a statistically significant result, 24-h ambulatory EEG recordings found statistical significance in electrographic seizure frequency reduction between the groups – with 8/10 patients in the stimulation on group versus 0/10 in the stimulation off group being responders. The median reduction in seizure frequency was 46.7% on diaries and 53.8% on EEG. Importantly, there was a reduction in seizure frequency in the stimulation off group once the group had activation of the DBS in the unblinded phase.

Although the *ESTEL Trial* did not meet its primary endpoint, with an underpowered analysis, it demonstrates a milestone in the development of neuromodulation strategies for patients with LGS and generalized-onset seizures and provides the first Level 1 evidence in this field. Interesting sub-analyses from the study have shown progress in the research of EEG-derived biomarkers of response to DBS therapy [31], which has implications for the improvement of therapy and strive toward adaptive DBS strategies [32].

There has not yet been a true RCT of DBS for paediatric epilepsy [33,34]. The closest endeavor was the *ADVANCE Trial (Add-On Deep Brain Stimulation versus Continued Vagus Nerve Stimulation for Childhood Epilepsy)* by Suresh et al. from *Toronto’s Hospital for Sick Children*. This was a single centre prospective clinical trial of DBS for children (8–18 years) who have been treated with but did not respond (<50% seizure frequency reduction) to VNS. A partially randomized approach was adopted, which allowed the parent/carer to choose between the addition of DBS or to continue with optimization strategies of VNS therapy. 7 patients received add-on DBS (Medtronic Percept DBS System), and 11 patients had continued VNS therapy. The DBS target was chosen according to the seizure-onset type - those with frontotemporal onset received ANT DBS and those with multifocal and generalized onsets received CMT DBS. The trial showed that the add-on DBS was superior in providing seizure frequency reduction at 1 year compared to VNS alone – mean of 51.9% vs 12.3%, respectively. Secondary outcomes determined that seizure severity was improved but overall quality of life was not different. The ADVANCE Trial had some methodological limitations – for example, it was a small-scale trial, it was unblinded, the patient group was heterogeneous and there is likely bias in the method of measuring seizure reduction (parental recall). However, this is an important clinical trial in paediatric neuromodulation for epilepsy – not only because it is the highest level of evidence in the field – but because of its novel approach to choose the DBS target according to the patient seizure-onset type. As will be discussed in the next section, this is a crucial next step for clinical trials in neuromodulation to overcome.

There have been smaller scale RCTs of DBS of non-thalamic intracranial targets. For example, the *Hips-DBS Trial* by Cukiert et al. was an RCT allocating patients to either active (n = 8) or inactive (n = 8) DBS of the hippocampi in patients with refractory TLE (determined on EEG and imaging) who were ineligible or declined temporal lobectomy [35]. The study found that 4/8 patients in the active group became seizure free and 7/8 were considered responders (>50% seizure frequency reduction) measured on seizure diaries.

### Responsive neurostimulation

Closed-loop intracranial neuromodulation is available in North America in the form of RNS (NeuroPace®) – a therapy that was designed specifically for patients with drug-resistant focal epilepsy not amenable to resection. Whereas in DBS there is typically a stereotyped rhythmical delivery of stimulation, RNS delivers stimulation to the target in response to ictal or inter-ictal epileptiform discharges [36]. The device has a skull-mounted implanted pulse generator (IPG), a sensing electrode (typically implanted either at the putative seizure onset zone or thalamic targets) and a stimulating electrode to deliver a closed-loop stimulation algorithm. Initial RNS set up was designed to respond to, and prevent, seizure propagation, but more recently setup has generally been optimized to respond to interictal fast activity and thus deliver frequent and long-term modulation of the epileptogenic network [[37], [38], [39]]. This therapeutic approach may provide similar effect to that of current DBS systems.

There have been three trials of RNS (1): an open-label feasibility (2): the Pivotal study by Morrell et al. [40]; and (3) a long-term outcomes study [41]. We here focus on the Pivotal trial of RNS, which was a double-blind, RCT. Following a 12-week baseline period, 191 adult patients (34.9±11.6 (range 18–66) years) underwent implantation of the RNS system and a four-week recovery period before being randomized to 12-weeks of stimulation (n = 97) on vs stimulation off (n = 94). The primary efficacy outcome showed mean % seizure frequency reduction was significantly higher in the treatment group (37.9%) when compared to the control group (17.3%).

The *Pivotal* RNS trial, reported by Morrell et al. in 2011, provided the first Class I evidence of intracranial closed-loop stimulation for the treatment of drug-resistant epilepsy. The nine-year, follow-up, open-label phase of the *Pivotal* trial and its preceding open-label study demonstrates that RNS provides durable clinical benefit as a long-term therapy [42].

### Epicranial neuromodulation

The *Epicranial Application of Stimulation Electrodes for Epilepsy* (EASEE®) neuromodulation system is a recent development in the field of neuromodulation for epilepsy and marks a major step forward in the development of extracranial strategies. The therapy is designed as minimally invasive for patients with focal-onset seizures. It requires a stimulation device to be implanted between the scalp and the skull at a location overlying the seizure onset. An IPG is implanted in the chest wall, akin to DBS or VNS systems.

The study published by Schulze-Bonhage et al. in 2023 reported the pooled results of two prospective, multicentre single arm, open label studies of the EASEE system [59]. After six months of active stimulation, the primary outcome was that 17 of the 32 were responders to the treatment (>50% seizure frequency reduction on seizure diaries). A two-year follow-up study of 22 of the participants showed that the responder rate improved to 65.4% [43]. Although this outcome was promising and comparable to intracranial neuromodulation therapy results, there is a notable limitation in that there was no control group, or blinding of the therapy.

## Current & Future Clinical Trials in Neuromodulation for Epilepsy

Despite advances in technology and these landmark clinical trials, the availability and utility of neuromodulation therapies are not fully adopted in all healthcare systems. Reasons for this uptake hesitancy are multifactorial, but include (a) clinical trials reporting modest efficacy results; (b) the need for refined therapy strategy (for example patient selection, target selection [26]; (c) parameter searching [46]); (d) device hardware/software refinement, particularly for paediatric populations [47,48]; and (e) consideration of device cost/economics [49]. Additionally, the landmark RCTs of neuromodulation for epilepsy have treated adults, and further robust trials are required in paediatrics – which has motivated the recently published *ADVANCE Trial* [28] and the upcoming *Children’s Adaptive Deep brain stimulation for Epilepsy Trial* (CADET) [50] (Table 3). Research for neuromodulation for epilepsy continues to progress to meet these challenges, and this final section of the review speculates on the future of clinical trials in this field.

**Table 3:** Ongoing clinical trials of neuromodulation therapies (vagus nerve stimulation (VNS), deep brain stimulation (DBS) and responsive neurostimulation (RNS)) for the treatment of epilepsy∗. Results are categorised according to VNS trials, DBS trials targeting the anterior nucleus of the thalamus (ANT), DBS trials targeting other brain targets, RNS trials, stimulation parameter trials, and paediatric trials. This table searches clinicaltrials.gov and is not an exhaustive list. ANT, anterior nucleus of the thalamus; DRE, drug-resistant epilepsy; CL, central lateral nucleus of the thalamus; CMT, centromedian nucleus of the thalamus; LGS, Lennox-Gastaut syndrome; NCT ID, ClinicalTrials.gov identifier; RCT, randomized controlled trial; STN, subthalamic nucleus; VNS, vagus nerve stimulation.

Trial [NCT ID]	Design	Primary objective	Key eligibility criteria (not exhaustive)	Target sample size	Device	Target	Status	Sponsor
**VNS trials**								
A first in human study for resistant epilepsy with the VNS device by synergia medical (AURORA) [NCT06340802]	• Multicentre (Belgium & Germany) • Single arm • First-in-human of device • Open label	• Safety of the device at 36-months follow-up	• Adults (≥18 years) • DRE	10	*NAO.VNS SYSTEM* (Synergia medical)	Vagus nerve	Not yet recruiting	Commercial
**DBS trials (ANT)**								
Medtronic DBS therapy for epilepsy post-approval study (EPAS) [NCT03900468]	• Multicentre (USA) • Open label • Single arm of stimulation therapy	• Seizure frequency reduction (patient diaries) at 36-months	• Focal-onset seizures • DRE • 6 or more seizures per month	140	Medtronic activa PC or percept PC	ANT	Recruiting	Commercial
**DBS trials (non-ANT)**								
Centrolateral/intralaminar thalamus stimulation to restore arousal in TLE (START) [NCT04897776]	• Multicentre (USA) • RCT • Blinded crossover design: Active vs inactive/sham arms	• Change in conscious awareness during and following seizures (behavioural responsiveness scale) up to 45 months	• Adults (≥18 years) • DRE • Mesial temporal lobe seizures • At least 2 seizures per month involving impaired awareness	5	Medtronic RC + S	Bilateral CL	Recruitment completed	Academic
Subthalamic nucleus stimulation for drug-resistant focal motor epilepsy (STEM) [NCT06248333]	• Multicentre (China) • RCT • Random allocation to active vs sham STN DBS	Seizure frequency reduction at 3-months of stimulation	• 14–65 years • DRE • Motor seizures • At least 3 seizures per month	33	Not defined	STN	Recruiting	Academic
Hippocampal + thalamic DBS for bilateral temporal lobe epilepsy [NCT04164056]	• Single center (China) • RCT • Open label • Allocation to hippocampal vs ANT stimulation	Responder rate, seizure free rate and seizure frequency reduction after 3 years	• 12–60 years • DRE • Bilateral temporal lobe epilepsy	80	Not defined	Hippocampus and ANT	Unknown	Academic
**RNS trials**								
RNS for idiopathic generalized epilepsy (NAUTILUS) [NCT05147571]	• Multicentre (USA) • RCT • Randomized to ON vs. OFF blinded phase followed by open-label phase	• Safety (serious device-related adverse events) • Time to second generalized tonic-clonic seizure (within 9-months)	• Age 12 years and older • DRE • EEG with features consistent with idiopathic generalized epilepsy • At least two generalized tonic-clonic seizures at baseline	80–100	RNS	Sensing electrode: Cortex. Stimulating electrode: CMT.	Closed to recruitment	Commercial
RNS system LGS feasibility study [NCT05339126]	• Multicentre (USA) • RCT • Randomized and crossover design: High-frequency vs. low-frequency vs. sham blinded phase followed by open-label phase	• Safety (serious device-related adverse events) • Responder rate (defined as ≥ 30% reduction of seizure frequency) during the blinded evaluation period	• Age 15 years and older (with potential to reduce to 12 years) • DRE • At least 5 drop seizures in the 2-months preceding enrolment • Non-localized seizures • EEG supporting diagnosis of LGS	24	RNS (two stimulators per patient)	Sensing electrodes on the left parieto-temporal cortex and right homologous region. Stimulating electrodes: Prefrontal cortex and CMT.	Closed to recruitment	Commercial
**Stimulation parameter trials**								
Optimization of DBS parameters in medically refractory epilepsy [NCT05493722]	• Single center (USA) • RCT • Crossover between 3 phases: Parameters decided by clinicians vs parameters optimized according to power spectral density measurements	• Difference in broadband power with different settings • Difference in frequency band specific lower frequency power with different settings	• DRE • Must have DBS device in-situ	20	Not defined	Not defined	Recruiting	Academic
Precision DBS programming with objective neural response (EPI-BOOST) [NCT06364085]	• Single center (Canada) • Open label • Device programming to be informed by physiological data from the DBS device	• Absolute seizure reduction at 12-months	• ‘Child’ to ‘older adult’ • DRE	40	Not defined	Not defined	Not yet recruiting	Academic
**Paediatric trials**								
Children’s adaptive deep brain stimulation for epilepsy trial (CADET) [NCT06924086]	• Multicentre (UK) • RCT • Randomized (active vs inactive) phase followed by open-label phase	• Seizure frequency reduction (diaries) at end of six months open label phase	• Age 5–14 years • Diagnosis of LGS • At least 10 seizures per month	22	Picostim® DBS system	CMT	Recruiting	Academic
Sensing, modulating and adapting in real-time in DBS (SMART-DBS) [NCT06924086]	• Multicentre (UK) • Open label • Single arm of adaptive DBS	• Feasibility of adaptive DBS and seizure sensing	• Age 5–17 years • Diagnosis of LGS • Prior implantation of the picostim device (CADET trial participants)	20	Picostim® DBS system with DyNeuMo-2 software [44,45]	CMT	Recruiting	Academic

Table 3 summarizes the clinical, interventional trials in VNS, DBS, RNS and EASEE that are registered as ongoing on clinicaltrials.gov as of the January 30th, 2026, omitting those who have already been published in the literature and discussed elsewhere. The accuracy of the status listed on clinicaltrials.gov has not been verified.

### Head-to-head neuromodulation trials

With multiple neuromodulation strategies emerging, how do clinicians and patients choose between the different treatment options available for focal-onset epilepsy? It appears that results across RCTs of neuromodulation therapies are broadly similar, however, comparison of these outcomes are currently indirect and are challenged by heterogeneity in patient selection, outcome measures and duration of follow up.

The challenges of running a head-to-head trial between neuromodulation therapies are acknowledged: (a) clinical trials are time-consuming, laboursome and expensive; (b) large trials are likely required to detect potentially small differences in effect size; (c) lack of equipoise between neuromodulation strategies may render patient recruitment difficult; (d) blinding patients between different neuromodulation therapies is essentially impossible; (e) patient groups within DRE are heterogeneous and such a trial would only likely answer the question for a particular type of DRE; (f) even within a single neuromodulation therapy, there are multiple variables (for example, stimulation parameters) that remain contentious before even considering comparison to another therapy; and (g) there is no financial incentive for manufacturers to fund a trial that may favour another therapy.

There is much progress to be made to refine each neuromodulation therapy before a head-to-head comparison can be definitively performed. For example, there are trials now underway to refine parameter setting within DBS therapies for epilepsy (Table 3). Furthermore, we would speculate that there is no *‘one-size-fits-all’* in terms of a single, neuromodulation strategy for all DRE. The development of several therapies with different features, advantages, disadvantages (and likely efficacy differences between groups of patients) will hopefully provide patients with tailored approaches and, most importantly, empower patients to choose the treatment that suits their needs and wishes.

### Trials of intracranial targeting in DBS/RNS

The *SANTÉ Trial* targeted the ANT for all patients with focal-onset seizures, regardless of the putative seizure onset zone [27]. Although the trial was not statistically powered to compare outcomes between seizure-onset zones, *SANTÉ* Trial found that seizure frequency reduction was significantly different between the stimulation and sham arms in patients with temporal and frontal epilepsies, but not in those with other seizure onset zones. There is a growing consensus that the *‘ANT DBS for all’* will not deliver adequate results and a shift toward personalised targeting based on features such as the seizure onset zone, patient syndrome, and presence/absence of focal-to-bilateral tonic-clonic seizures will be required. Although ANT DBS seems to be a reasonable strategy for patients with a confident diagnosis of limbic epilepsy, there is no clear algorithm for DBS/RNS target selection for patients with focal DRE with non-limbic seizure-onset zones/networks.

New targets are emerging and being trialed – such as the recent *PULSE Trial* (pulvinar DBS for focal epilepsy) [25], the *START Trial* (targeting the central-lateral nucleus for TLE and seizures with impaired awareness), the hippocampus [35], and the STEM Trial (subthalamic nucleus targeting for motor seizures) (Table 3). Even within an agreed nucleus there is growing evidence that subnucleus specificity may be required for optimal outcomes in DBS for DRE. ‘Sweet spot’ analyses from ANT studies suggest that proximity to the mammillothalamic tract is associated with greater seizure frequency reduction [51], supporting the hypothesis that modulation of the Circuit of Papez is a key therapeutic mechanism in DBS for limbic epilepsies [8]. Similarly, in CMT DBS for LGS, sweet spot analyses suggest that there is a subregion of the CMT (anterior and inferolateral) that is optimal for achieving better seizure frequency reduction [52]. Similar challenges as described above for head-to-head treatment modalities will present themselves for running clinical trials of head-to-head targeting approaches.

A potential route of reconciliation and navigating targeting options for focal epilepsy is the recent introduction of thalamic stereo-EEG in epilepsy surgery, with the premise that the derived electrophysiological markers are able to reconcile target selection for DBS/RNS. Multiple studies have demonstrated that thalamic stereo-EEG is safe and acceptable [53,54], but it remains unclear whether an EEG abnormality detected in a particular thalamic nucleus equates to identification of the optimal stimulation target [55]. Future trials of target selection and stereo-EEG guided target selection are required and underway [56], but robust evidence will require potentially complex clinical trials, large cohorts or adaptive clinical trial designs.

### Adaptive neuromodulation

Open-loop neuromodulation refers to the delivery of neuromodulation to the brain in the absence of a method to adapt the delivered therapy according to (a) physiological feedback (for example, epileptiform activity on EEG, or ictal tachycardia on electrocardiography) or (b) a marker of efficacy (e.g. clinical seizure onset). Closed-loop stimulation is already adopted in part by both VNS and RNS, but not yet in DBS. More recent evidence from RNS studies questions whether RNS is instead providing long-term neuromodulation to the epileptogenic network, akin to DBS, rather than having an acute effect on seizure propagation [39]. Further trials, such as the upcoming *Sensing, Modulating and Adapting in Real* Time (SMART-DBS) Trial, to investigate the additional benefit of adaptive stimulation are required (Table 3).

### Outcome measures

As discussed earlier in this article, the primary outcome for most neuromodulation trials in epilepsy has been seizure frequency reduction or responder rate. Although this is a highly measurable factor, it may not be the only factor that is important. Future trials should prioritize other factors that are important to patients with epilepsy and include iterative patient/public/stakeholder involvement in the design of these trials. Other important outcome measures include quality of life, impact on parents/carers, neurodevelopmental outcomes (particularly in children), and mental health. Economics is also an important factor and, for example, an emerging study from the UK by Marson et al. [60] will repeat a multicentre RCT with the objective of investigating the economic value of VNS for patients with epilepsy.

Lastly, given our insight from VNS data that suggests that seizure frequency reduction improves with time, clinical trials should include the means to track data for several years to robustly study the outcomes of neuromodulation therapies for epilepsy over the long-term.

## Conclusions

The landmark RCTs of neuromodulation therapies (namely VNS, DBS and RNS) have demonstrated the potential meaningful benefits to patients with DRE. Current and future trials will include investigating non-ANT brain targets, refining targeting strategies, defining the added benefit of adaptive stimulation approaches, and exploring the stimulation parameter space in neuromodulation strategies for epilepsy. The major challenges in translation that lie ahead are (a) to maximize the impact that each therapy can provide; and (b) to develop the knowledge to provide precision strategies of neuromodulation to maximize outcomes for the individual patient.

## Funding

RJP and MMT are funded by the National Institute for Health and Care Research (NIHR). RJP is funded by GOSH Children's Charity.

## Author contributions

Conceptualisation – RJP, MMT. Writing of manuscript – RJP. Review and approval of manuscript – RJP, MMT.

## Declaration of competing interest

No conflicts of interest.
